# Biotic soil-plant interaction processes explain most of hysteric soil CO_2_ efflux response to temperature in cross-factorial mesocosm experiment

**DOI:** 10.1038/s41598-019-55390-6

**Published:** 2020-01-22

**Authors:** Yann Dusza, Enrique P. Sanchez-Cañete, Jean-François Le Galliard, Régis Ferrière, Simon Chollet, Florent Massol, Amandine Hansart, Sabrina Juarez, Katerina Dontsova, Joost van Haren, Peter Troch, Mitchell A. Pavao-Zuckerman, Erik Hamerlynck, Greg A. Barron-Gafford

**Affiliations:** 1 Centre de recherche en écologie expérimentale et prédictive (CEREEP-Ecotron IleDeFrance), Département de biologie, Ecole normale supérieure, CNRS, PSL University, 77140 St-Pierre-les-Nemours, France; 2 Departamento de Física Aplicada, Universidad de Granada, 18071 Granada, Spain; 3 Sorbonne Université, CNRS, Institut d’Écologie et des Sciences de l’Environnement de Paris (iEES-Paris), Faculté des Sciences et Ingénierie, 75005 Paris, France; 4 Institut de Biologie de l’Ens (IBENS), Département de biologie, Ecole normale supérieure, CNRS, PSL University, 75005 Paris, France; 5 Department of Ecology and Evolutionary Biology, University of Arizona, Tucson, Arizona 85721 United States; 6 Biosphere 2, Office of Research, Development, & Innovation, University of Arizona, Tucson, Arizona 85721 United States; 7 Department of Hydrology & Atmospheric Sciences, University of Arizona, Tucson, Arizona 85721 United States; 8 Department of Environmental Science and Technology, University of Maryland, College Park, Maryland 20742 United States; 9 US Department of Agriculture-Agricultural Research Service, Eastern Oregon Agricultural Research Center, Burns, OR 97720 United States; 10 School of Geography & Development, University of Arizona, Tucson, Arizona 85721 United States

**Keywords:** Carbon cycle, Carbon cycle

## Abstract

Ecosystem carbon flux partitioning is strongly influenced by poorly constrained soil CO_2_ efflux (*F*_*soil*_). Simple model applications (Arrhenius and Q_10_) do not account for observed diel hysteresis between *F*_*soil*_ and soil temperature. How this hysteresis emerges and how it will respond to variation in vegetation or soil moisture remains unknown. We used an ecosystem-level experimental system to independently control potential abiotic and biotic drivers of the F_soil_-T hysteresis. We hypothesized a principally biological cause for the hysteresis. Alternatively, *F*_*soil*_ hysteresis is primarily driven by thermal convection through the soil profile. We conducted experiments under normal, fluctuating diurnal soil temperatures and under conditions where we held soil temperature near constant. We found (*i*) significant and nearly equal amplitudes of hysteresis regardless of soil temperature regime, and (*ii*) the amplitude of hysteresis was most closely tied to baseline rates of *F*_*soil*_, which were mostly driven by photosynthetic rates. Together, these findings suggest a more biologically-driven mechanism associated with photosynthate transport in yielding the observed patterns of soil CO_2_ efflux being out of sync with soil temperature. These findings should be considered on future partitioning models of ecosystem respiration.

## Introduction

A major challenge in terrestrial carbon science is identifying atmospheric CO_2_ source and sink dynamics across numerous timescales^1–3^. Because CO_2_ efflux (*F*_*soil*_) can be the largest and most variable component flux in many ecosystems^4^, *F*_*soil*_ drives regional carbon dynamics^5,6^. Accurate measurements of *F*_*soil*_ are critical for partitioning net ecosystem CO_2_ flux (*NEE*) and modeling local-to-global carbon dynamics^4,7,8^. Nighttime ecosystem respiration (*R*_*eco*_) can be un-measurable using the eddy covariance (EC) technique because of a lack of turbulence and atmospheric mixing, requiring gap-filling procedures to produce credible sums^7^. These missing nighttime and all-daytime estimates of *R*_*eco*_ are often approximated using an exponential temperature (T)*-R*_*eco*_ regression model derived from nighttime EC data and *T*, which assumes that the diel temperature sensitivity can be fit using an Arrhenius or Q_10_ model^8–10^. Incorrect daytime *R*_*eco*_ confounds *NEE* partitioning into *R*_*eco*_ and gross ecosystem productivity (*GEP*). Therefore, an incorrect model to estimate *R*_*eco*_ leads to greater uncertainty and bias in local-to-global estimates of NEE and its components. Some have suggested that the primary factors limiting our ability to characterize soil carbon metabolism and CO_2_ efflux include lags and antecedent features of abiotic and biotic drivers associated with above- and belowground processes^10–14^.

Considerable progress has been made in *F*_*soil*_ modeling by moving beyond simple temperature response functions (see^15^) to developing frameworks that account for multiple vegetative cover types or soil microhabitats^16–19^ and incorporate the important, but variable, influence of antecedent environmental effects^20,21^ and biotic inputs^14^. Still, our limited understanding of abiotic (e.g., environmental) and biotic (e.g., aboveground plant function) interactions constrains robust modeling of *F*_*soil*_^12,22–24^. Many studies have shown that Arrhenius^15^ or Q_10_^25^ functions poorly describe temperature dependence of *F*_*soil*_ – globally, and regardless of ecosystem type. Instead, *F*_*soil*_ often demonstrates a hysteretic response with temperature^11,26–42^. In a hysteretic relationship, the dependent variable can be at multiple states for a given value of the explanatory variable depending on the system’s history. Here, this means that a model based on a *T* (the explanatory variable) can give you two different estimates of *F*_*soil*_ (the dependent variable). How, then, can we expect numerical modeling to capture and appropriately predict rates of this dominant carbon source to the atmosphere, when we know the primary numerical relationship is so flawed? Both biological and physical processes can cause this hysteresis. Biological processes driving *F*_*soil*_ hysteresis might include patterns of photosynthate allocation^12,14,33^, physiological upregulation^43^, phenology^13,14^, soil water redistribution^21,44,45^, and dynamic storage and loss of carbon in response to micrometeorology^27,36,46–50^.

Physical processes contributing to observed decoupling of *F*_*soil*_ from *T* include heat transport and gas diffusion through the soil^51^. For example, hysteresis can increase with soil drying because of decreased thermal diffusivity^29,33^, or increase with soil wetting because of decreased gas diffusivity^52^. While a lack of observed *T-F*_*soil*_ relationships under field conditions does not negate theories of biological or enzymatic temperature dependence^23^, hysteresis complicates prediction of surface CO_2_ efflux and requires improved model formulations. Previous efforts to distinguish these physical processes from the influence of biological substrate inputs have been hindered, in part, by the lack of an ability to control meso-scale temperature of a soil column that could differentially regulate these contributing drivers.

The central question we address here is: what is the relative contribution of biotic and abiotic drivers in determining the hysteretic relationship between *F*_*soil*_ and temperature and how do these contributions vary across environmental gradients of moisture? We explore this question under semiarid conditions with semiarid vegetation. Semiarid regions experience multiple wet-dry transitions that create simultaneous ‘pulses’ of biological activity and alterations to the physical characteristics of the ecosystem^45^– making them an ideal setting for attempting to detangle these biotic and abiotic drivers. We hypothesized that lag in the delivery of recent photosynthate to soil leads to a hysteretic relationship between *F*_*soil*_ and temperature and that this lag increases with increased input from the plant (associated with the leaf area and net photosynthetic rates). In fact, previous studies have found this hysteretic relationship to be more prominent under woody plants than under grasses or bare soils^33^. As such, wetter conditions, which are likely to stimulate photosynthate production and transport, might induce greater hysteresis in the relationship between *F*_*soil*_ and temperature. The alternate hypothesis is that hysteresis follows the decoupling of soil *T* relative to *F*_*soil*_, not stimulation of *F*_*soil*_ by photosynthate. As noted by Phillips *et al*.^51^, the hysteretic response is likely due to the influence of both of these factors.

How biotic and abiotic drivers modulate patterns of *F*_*soil*_ in the context of mixed vegetation ecosystems is difficult to assess because of the potential, and variable, role they may play in driving fluxes. Therefore, the objectives of this study were to (*i*) quantify the response of *F*_*soil*_ to temperature, soil water content, and leaf-level carbon gain in a suite of controlled mesocosms and (*ii)* determine the relative influence of abiotic (soil temperature and soil water content) and biotic (photosynthesis) factors on *F*_*soil*_ rates and the amplitude of any hysteretic response to soil *T* across a range of moisture conditions. Within each mesocosm, we monitored air temperature, soil temperature, soil moisture, and soil CO_2_ concentration continuously for two-week periods. At the same time, we measured rates of net photosynthesis of the vegetation to assess aboveground patterns of productivity. Then, we integrated these datasets. Because we wanted to understand the role of vegetative structure in determining the hysteretic relationship between T and *F*_*soil*_, we repeated these suites of measurements across mesocosms that contained (*i*) bare soil, (*ii*) bunchgrass, (*iii*) woody plants, or (*iv*) a mixture of both bunchgrass and woody plants.

## Results

### Environmental control and mesocosm vegetative development

**Figure 1:**
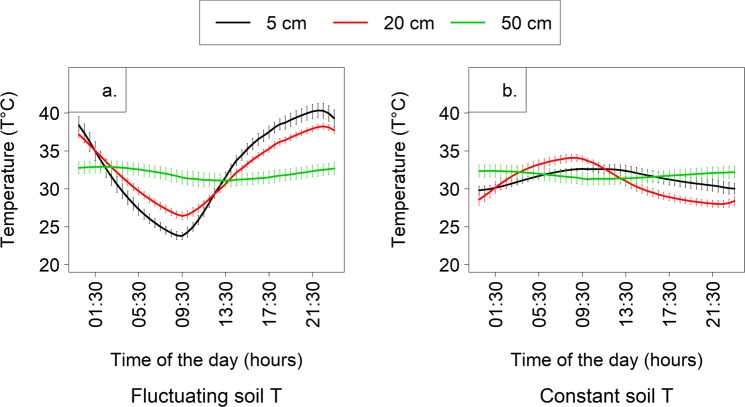
Average diel soil temperatures 5, 20, and 50 cm depths within Ecolabs set to mimic a (**a**) typical, fluctuating diurnal pattern and (**b**) relatively constant soil temperatures. Atmospheric CO_2_ concentrations and diel patterns of air temperature and the timing and intensity of light remained similar between these two treatments.

Tight regulation of soil temperature allowed us to create contrasting treatments in terms of a fluctuating diel pattern in soil temperature (30.88 ± 8.17 °C, representative of natural patterns; Fig. 1a) versus one with near-constant, modulated conditions (29.80 ± 2.43 °C; Fig. 1b). Similarly, precise irrigation yielded significant differences in treatment conditions between well-watered and dry soil moisture states (at 5 cm, 19.91 ± 0.57 versus 6.89 ± 0.37%, respectively).

The two vegetation types utilized here are the grass *Bouteloua curtipendula* and the woody plant velvet mesquite (*Prosopis velutina* Woot.), representing fundamentally different growth forms. The grass is shallow-rooted, but produces a dense network of roots that occupy the upper ~30 cm of soil. The woody mesquite utilizes a network of shallow and deeper roots. As such, the grass has a significantly greater mass of fine and coarse roots driving near-surface soil CO_2_ efflux than does mesquite (p < 0.001; grass and mesquite root biomass averaged 24.15 ± 6.49 and 2.65 ± 1.30 g, respectively; see Supplementary Table 1).

### Relationship between diel soil CO_2_ efflux and soil temperature, soil moisture, and aboveground plant function

**Figure 2:**
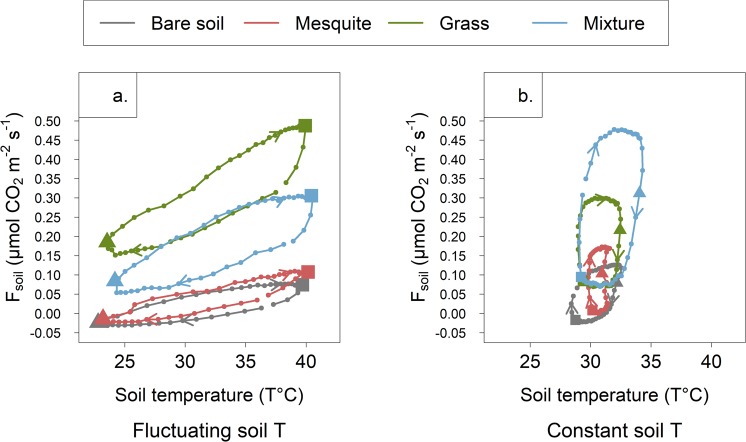
Average diel soil CO_2_ efflux plotted against average soil temperature for all biotic treatments within soil temperature treatments. Lights were turned on at 09:30 (represented by a triangle) and shut off at 21:30 (represented by a square). Arrows represent the clockwise hysteresis detected in all situations. *F*_*soil*_ was estimated from 5 cm depth to the surface. Wet and dry conditions are computed together for each soil temperature treatment.

Throughout the experiment, we observed the same elliptical shape and clockwise direction in the hysteretic relationship between soil temperature and *F*_*soil*_ for each vegetation type, regardless of the imposed temperature treatment (Fig. 2). Only the amplitude of hysteresis differed, with higher values associated with mesocosms that contained grasses. Because we experimentally constrained the diel range of temperatures experienced within the “constant” treatment, the X-axis is confined to a 5 °C band, but the range of *F*_*soil*_ we detected did not change. Soil moisture did not affect general hysteretic patterns (Supplementary Fig. 1). Rates of *F*_*soil*_ are very low at 20 and 50 cm depths – beyond the primary rooting depths of the plants in this experiment, underscoring a strong influence of vegetation on *F*_*soil*_ (Supplementary Fig. 2).

**Figure 3:**
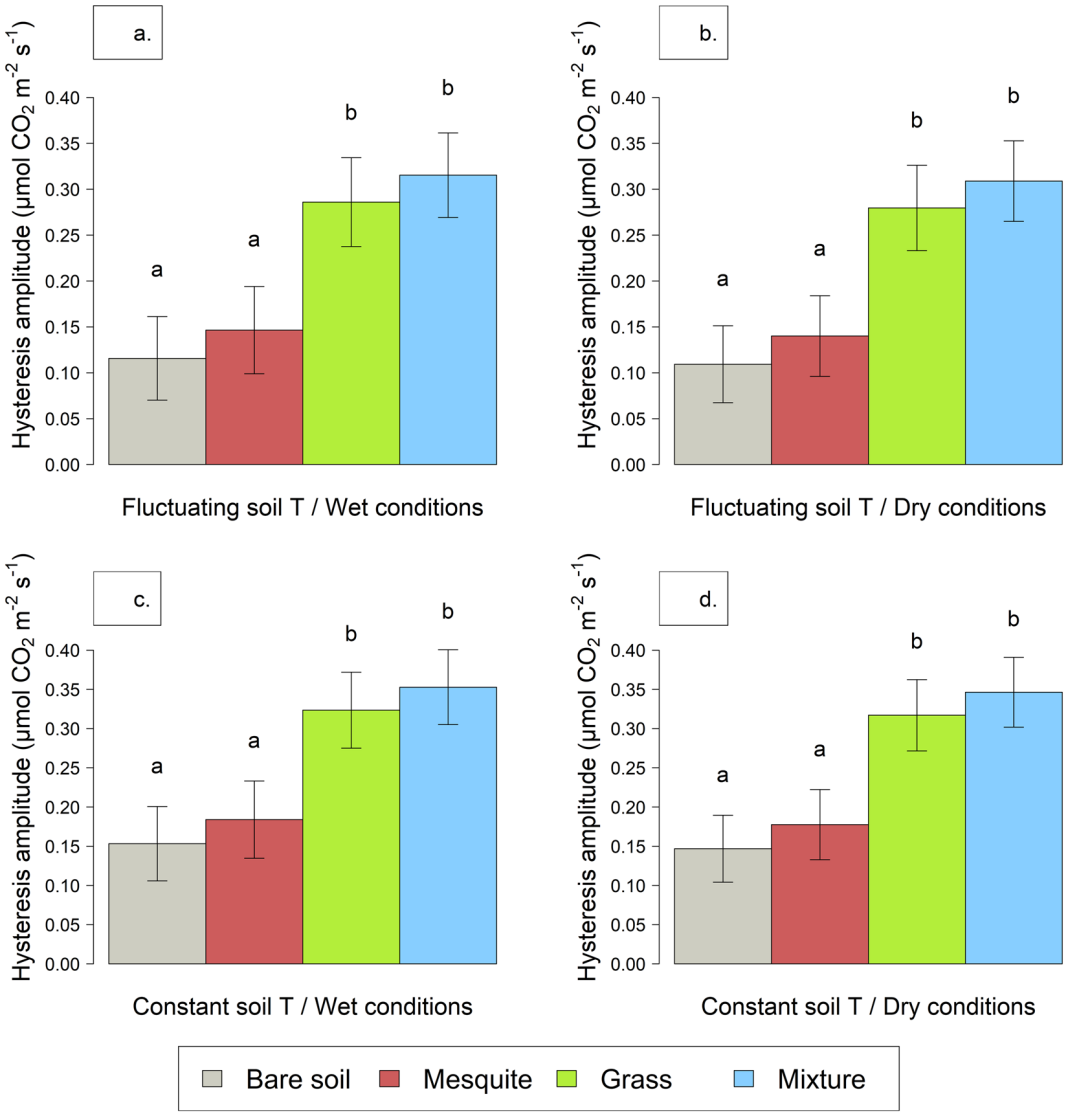
Adjusted mean of hysteresis amplitude (±standard errors) as a function of soil temperature across temperature treatments (fluctuating versus constant), targeted watering conditions (wet versus dry), and vegetation types. Letters indicate differences (p < 0.05) within each treatment.

Differences in vegetation were the only significant driver of variation in the amplitude of the hysteretic relationship between soil temperature and *F*_*soil*_ (R^2^ = 0.49; p < 0.001; Fig. 3), and the presence of grasses always increased the hysteresis amplitude. We did not detect any statistical difference between the amplitudes of the hysteresis due to the imposed temperature treatment (Table 1, Fig. 3 top versus bottom panels) or targeted watering conditions (wet versus dry; Fig. 3 left versus right panels, respectively).

**Table 1:** R^2^, degrees of freedom, F and p-values for ANOVAs performed on the fitted model for the amplitude of hysteresis as a function of soil temperature, soil moisture and vegetation type. Non-significant interactions are not shown. R^2^m and R^2^c stand for marginal and conditional squared-R, respectively.

Model		ANOVA degrees of freedom/ F-values/significance			
R^2^m	R^2^c		Soil Temperature	Soil Moisture	Vegetation type
0.40	0.49	DF (num.den)	1.44	1.44	3.44
		F-value	1.64	0.99	10.66
		p-value	0.21	0.33	<0.0001

**Figure 4:**
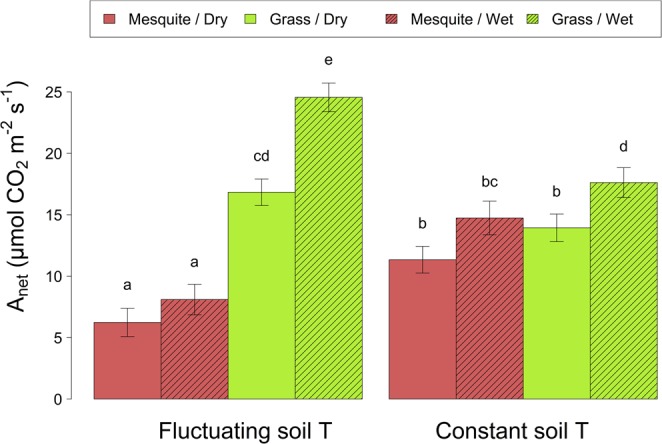
Adjusted mean of *A*_*net*_ (±standard errors) for grasses and mesquites grown in monoculture as a function of soil temperature (soil T) and moisture treatments. Lower case letters indicate differences (p < 0.05). Average is based on *A*_*net*_ measured from 11:30 to 19:30.

Average rates of net photosynthesis (*A*_*net*_) were in most cases greater in the grasses than in the mesquites (Table 2; Fig. 4), although grasses in constant soil temperature and dry conditions did not reach higher photosynthesis levels than mesquites under the same conditions. Though both vegetative forms responded positively to wet versus dry conditions in terms of their average *A*_*net*_, bunchgrasses were more significantly stimulated by the wet conditions.

**Table 2:** R^2^, degrees of freedom, F statistics and p-values for ANOVAs performed on the fitted model for *A*_*net*_ as a function of soil temperature (S.T), soil moisture (S.M) and plant species grown in monoculture (SpS). R^2^m and R^2^c stand for marginal and conditional squared-R, respectively.

Model		ANOVA degrees of freedom/ F-values/significance							
R^2^m	R^2^c		S.T	S.M	SpS	S.T* S.M	S.T* SpS	S.M* SpS	S.M *S.T *SpS
0.51	0.61	DF (num.den)	1. 804	1. 804	1. 804	1. 804	1. 804	1. 804	1. 804
		F-value	171.56	27.10	197.85	45.34	135.88	15.72	6.24
		p-value	<0.0001	<0.0001	<0.0001	0.0029	<0.0001	0.0001	0.0127

When pooling both vegetation types, we detected a significant positive relationship between rates *A*_*net*_ and soil respiration (*F*_*soil*_; R^2^ = 0.29; p < 0.0001) using the simple model: soil CO_2_ efflux = *f* (photosynthesis). However, this relationship was largely driven by the high rates of *A*_*net*_ and *F*_*soil*_ within bunchgrasses (R^2^ = 0.43; p = 0.0046), as there was no relationship within the mesquite mesocosms (R^2^ = 0.059; p = 0.3036).

**Figure 5:**
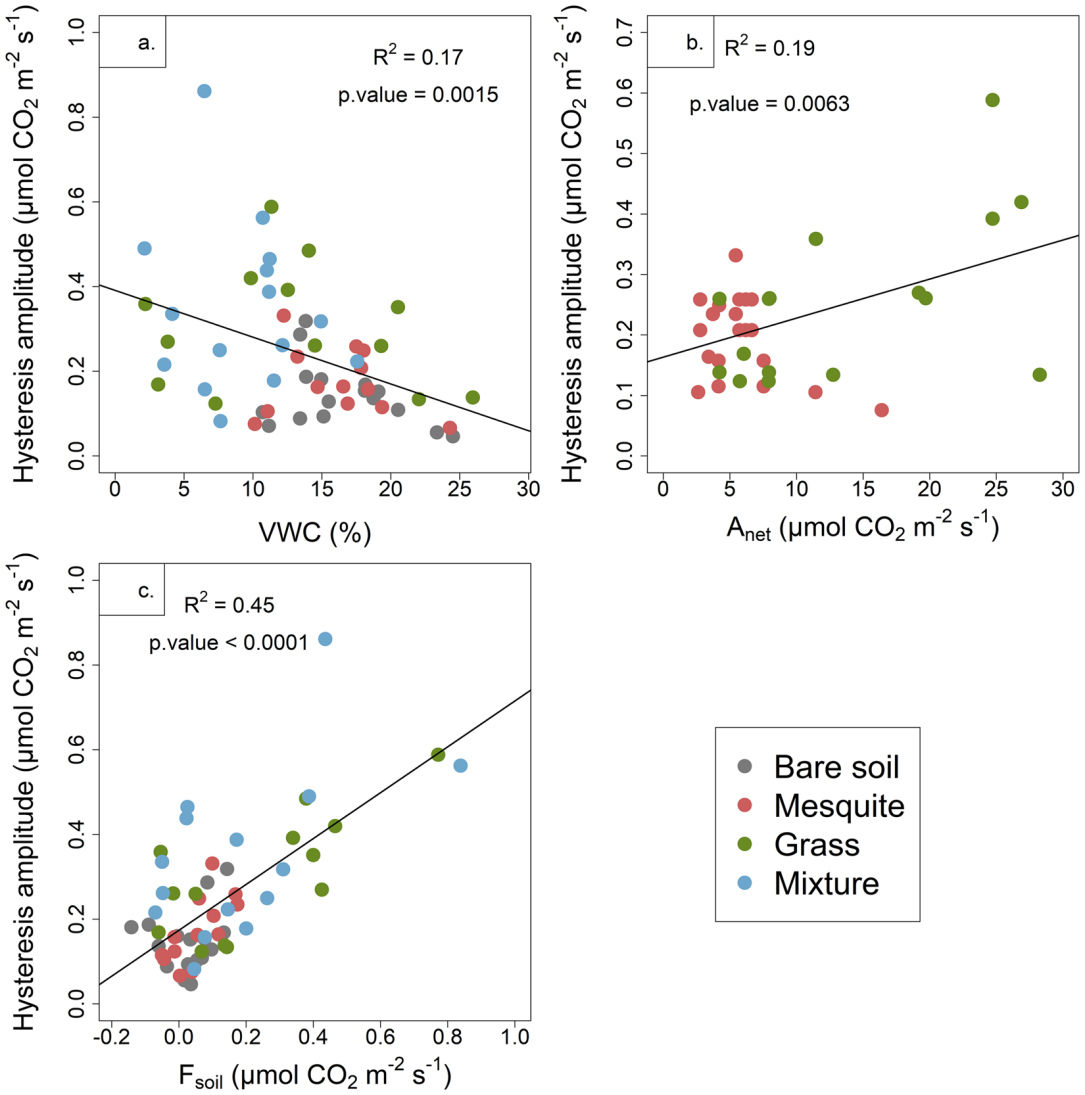
Illustrations of the linkages between the amplitude of hysteresis and (**a**) volumetric water content (VWC), (**b**) rates of net photosynthesis (*A*_*net*_) in monocultures and (**c**) rates of *F*_*soil*_.

Across all vegetative types, baseline rates of *F*_*soil*_ explained the most variation in the amplitude of the hysteretic relationship between soil temperature and *F*_*soil*_ and had the most significant correlation of all potential drivers of the hysteresis (Fig. 5). Volumetric water content (VWC) explained the least amount of variation in the amplitude of the hysteretic relationship between soil temperature and *F*_*soil*_, but we still detected a negative correlation when pooling across all vegetative cover types (Fig. 5a). However, when we examined the influence of VWC by species, we found no correlation with the amplitude of the hysteretic relationship (bare soil: R^2^ = 0.12, p = 0.148; mesquite: R^2^ = 0.03, p = 0.5496; grass: R^2^ = 0.03, p = 0.5744; and mixture: R^2^ = 0.02, p = 0.6344). Rates of Anet, an indirect driver of Fsoil, had the next most significant correlation, but the effect was species specific (Fig. 5b). We found a positive correlation between the hysteresis amplitude and Anet within grass (R^2^ = 0.29, p = 0.0270), but not within mesquite treatments (R^2^ = 0.18, p = 0.059). We found that the positive correlation between the hysteresis amplitude and *F*_*soil*_ was present across all vegetative types - grass (R^2^ = 0.54, p = 0.0040), mesquite (R^2^ = 0.49, p = 0.0081) and mixture (R^2^ = 0.31, p = 0.0031) - but not within bare soil treatments (Fig. 5c).

## Discussion

### Are hysteretic patterns between CO_2_ efflux and soil temperature driven more by biotic or abiotic properties?

The ways in which biotic and abiotic drivers differentially determine hysteretic patterns between *F*_*soil*_ and temperature have been difficult to assess because of the potential, yet variable, roles they may play in driving rates of soil CO_2_ efflux. We used controlled conditions to isolate abiotic from biotic drivers and simultaneously measured rates of aboveground photosynthetic assimilation to infer carbon source dynamics responsible for yielding different rates and temporal patterns of *F*_*soil*_. Our results support a more biologically-driven mechanism associated with photosynthate transport in yielding the observed patterns of soil CO_2_ efflux being out of sync with soil temperature. This assertion is supported by two key findings. First, we found significant and nearly equal amplitudes of clockwise hysteretic behavior between *F*_*soil*_ and soil temperature whether we allowed diel patterns of soil temperature to follow a typical sinuous curve or we held soil temperatures relatively constant. This finding is contrary to the alternative hypothesis that the hysteretic pattern stems from the differential propagation of heat through the soil profile and CO_2_ diffusion because we found the same pattern behavior when there was no heat propagation through the soil. Second, we found that the amplitude of hysteresis between *F*_*soil*_ and soil temperature was most strongly tied to baseline rates of *F*_*soil*_, which is strongly driven by the amount of fine root biomass. The majority of the residual relationship is tied to aboveground biological inputs through rates of net photosynthesis.

Others had previously hypothesized this biological driver of the hysteretic behavior based on their documentation of the phenomenon and concurrent measurements of photosynthetic rates, but no study to date had isolated photosynthetic fluxes and abiotic drivers as directly as here. For example, Vargas and Allen^31^ noted a relationship between *F*_*soil*_ and soil temperature that resulted in variable rates of photosynthesis in the overstory and understory vegetation under a range of natural conditions. Similarly, Barron-Gafford *et al*.^6^ found that the degree of hysteresis was positively correlated with photosynthetic rates of the overstory in a semiarid savanna. Importantly, the hysteresis observed here was greatest in mesocosms occupied by grasses. Previous research has illustrated a very short lag in the time between carbon assimilation until stimulation of *F*_*soil*_, ranging from hours^50^ to ~1 day^14,27^. These studies, then, would suggest that same-day and day-prior photosynthesis rates were most important in determining current-day *F*_*soil*_ under bunchgrasses^13,14,21^. Longer lag times, presumably due to longer phloem transport distance, within mesquite would reduce the correlation between these concurrent fluxes. This may explain the positive correlation we found between *A*_net_ and *F*_*soil*_ for bunchgrass mesocosms, but the decoupling between *A*_*net*_ and *F*_*soil*_ for mesquite mesocosms in this study.

### How do contributions to the hysteretic patterns between CO_2_ efflux and soil temperature vary across different plant types and environmental gradients?

Rates of net photosynthesis per unit leaf area were greater in the bunchgrasses than in the mesquite. Likewise, total leaf biomass in the bunchgrass mesocosms was six times greater than in the mesquite, and total root biomass in the bunchgrass mesocosms was nine times greater than in the mesquite. Together, these factors would yield significantly greater total photosynthate input into the soils of the mesocosm that contained bunchgrass than those that contained mesquite. This positive relationship between aboveground carbon inputs (rates of net photosynthesis) and *F*_*soil*_ is expected given that *F*_*soil*_ is the result of autotrophic and heterotrophic source of soil respiration (as recently summarized by Song *et al*.^36^).

Differences in net photosynthetic rates in mesquite and grass tend to depend on moisture conditions, with grass having higher rates under wet conditions and mesquite having similar rates under dry and wet conditions because of the rooting strategies of mesquite that allow for greater access to deep water^14,45,53,54^. However, these differences in net photosynthetic rates are dependent on the size and age of the woody plant, with smaller mesquites often experiencing significantly lower rates of carbon assimilation than larger individuals^53,55–57^. Thus, the lower photosynthetic rates in mesquite than in bunchgrass found here are in line with previous research, when considering that the mesquites were less than one year old at the time of the experiment.

The small hysteretic pattern between *F*_*soil*_ and soil temperature within the bare soil mesocosm might be surprising given that there is no vegetation to deliver photosynthetic products. However, previous research has illustrated that in dryland and Mediterranean ecosystems with alkaline soils, there can be a chemical process of carbonate precipitation and dissolution^36,58–66^. The resulting periods of CO_2_ absorption removal would contribute to a hysteretic pattern between *F*_*soil*_ and soil temperature because for the same range of temperatures one can detect daytime net CO_2_ efflux, but nighttime net CO_2_ influx from the atmosphere due, in part, to strong soil-air temperature gradients. The soils used here, however, contained little inorganic carbon. Even in our more strongly constrained temperature regime, some propagation of temperatures still occurred, and we suggest that it may have affected the small amount microbial activity present and likely drove some of the inorganic processes. As such, the patterns seen in the bare soil treatment likely illustrates the concomitant influence of abiotic and biotic drivers.

## Conclusion

The use of precise climatic and soil condition controls allowed us to test whether the hysteretic relationship between *F*_*soil*_ and soil temperature was mainly driven by abiotic or biotic conditions. We observed a strong influence of biotic factors on hysteretic behaviors. We suggest that the delivery of photosynthate in the soil is a major factor in creating lag in the relationship between soil CO_2_ efflux and soil temperature. In particular, the high photosynthetic rate and biomass of bunchgrass was associated with higher baseline rates *F*_*soil*_ and hysteresis amplitudes. Therefore, variation in plant community structure likely has an important regulatory role in governing how *F*_*soil*_ responds dynamically to climate drivers, with potentially profound impacts on seasonal ecosystem-level respiration rates.

## Methods

### Experimental facility and environmental monitoring

The experiment was conducted at the Ecotron Île-de-France facility (St-Pierre-les-Nemours, France), which houses a suite of highly controllable meso-scale ‘Ecolabs’. Each Ecolab permits the simultaneous manipulation of multiple atmospheric parameters and climatic variables across three individual 13 m^3^ chamber (see Verdier *et al*.^67^ for extensive technical descriptions and Supplementary Methods 1 for pictures). Within each of these chambers, there is a 1 m tall lysimeter with 1 m^3^ volume in which we placed four separate 60 cm tall mesocosms (0.07 m^3^ volume). Mesocosms were either left with bare soil or planted with the woody plant velvet mesquite (*Prosopis velutina* Woot.) only, the grass *Bouteloua curtipendula* only, or a mixed community of *P. velutina* and *B. curtipendula*. In total, six chambers and 24 mesocosm were used. We used a loamy sand-textured basalt with a porosity of 37% and bulk density of 1.5 g cm^−3^ as our soil matrix. The soil had an inorganic carbon content of 2.30 × 10^−5^ g g^−1^, and a pH of 8.18; further details on the soil chemistry are previously reported^68,69^. *P. velutina* and *B. curtipendula* seeds used in this study originated from a site located in the Santa Rita Experimental Range (31.8214°N, 110.8661°W, elevation: 1116 m) south of Tucson, Arizona, USA. This area was historically a grassland, but is now dominated by *P. velutina*, which covers approximately 35% of the ~2800 m^2^ study site. Much of the *P. velutina* understory and intercanopy space consists of a mosaic of bunchgrasses, including *B. curtipendula*, *Eragrostis lehmanniana* Nees, *Digitaria californica* Benth, and *B. eriopoda*. Mean annual precipitation at this site is 375 mm, with about 50% falling in July-September as part of the North American Monsoon. Scott *et al*.^3^ described additional details on the site. We set up an establishment phase of 4 months to allow the plants to grow before the measurements. In mesocosms that include grass, 4 g of seeds were sown. As for mesquites, 30 seeds were initially sown in each mesocosm. After a month, plants were thinned to only three mesquites per mesocosm.

We monitored atmospheric CO_2_ concentration ([CO_2_]) using a flow-through loop linking each Ecolab chamber in-line to a gas analyzer (LI-840; LI-COR, Lincoln, Nebraska, USA). Precise control of atmospheric [CO_2_] was maintained by a solenoid valves that allow for direct injection and by CO_2_ absorption using soda lime when necessary. We set [CO_2_] and air relative humidity at 400 ppm and 30%, respectively. Along with measures of air temperature and relative humidity, air samples were measured automatically every 30 seconds, and an average for each chamber within each Ecolab was recorded every 30 minutes. Further, we monitored soil moisture (MAS-1; Decagon Devices Inc., Pullman, WA, USA) and temperature (PT-100) at the near surface (5 cm) and at 20 cm and 50 cm depths within each replicate mesocosm. Again, measurements were conducted every 30 seconds, and we recorded an average for each mesocosm every 30 minutes. We delivered light by an overhead plasma lamp (Lumixo-A, Spectrum AM 1.5, Bulb M46, Lumartix, Aubonne, Switzerland) with a 12-hour day length that was set to occur between 09:30 and 21:30 local time.

### Experimental design controlling above and belowground temperatures and soil moisture conditions

We independently controlled above- and belowground temperatures, systematically targeting the role of abiotic versus biotic drivers of hysteretic patterns in *F*_*soil*_. We repeated the following pair of environmental cycles under wet and dry soil moisture conditions: (*i*) a pattern of diel aboveground and soil temperature cycles, which mimics natural conditions of the home field site and serves as a control treatment and (*ii*) a pattern of diel cycle aboveground temperature but constant soil temperature. This treatment constrains vertical soil temperature gradients, a primary hypothesized abiotic driver of the hysteretic relationship between *F*_*soil*_ and soil *T*, while mimicking natural aboveground conditions. To reduce the amplitude of temperature variation at the surface, pipes surrounding the mesocosm surface were filled with an antifreeze liquid either to heat-up or to cool-down the system. The surface of the lysimeter was constantly maintained at 33 °C, and the bottom of the mesocosm was allowed to stabilize through heat transfer. In constant soil temperature conditions, soil cooling occurred during the daytime, whereas warming occurred during nighttime, leading to desynchronized light patterns, air temperature, and soil temperature patterns. To simulate wet conditions, mesocosms received 5 mm of tap water twice a week using a dripping irrigation system that allowed for a slow release of water into the soil. We did not add any water during the ‘dry’ treatments to achieve dry soil moisture conditions. In order to dampen potential legacy effects of individual treatments through time experience for each of the mesocosms, we (*i*) randomized the timing of each treatment for each mesocosm, (*ii*) introduced a transition period of one week between each treatment in which the mesocosm went through the new soil moisture and soil temperature settings to allow for acclimation to the new conditions, and (*iii*) we ran each treatment for a two-week period. This experimental plan yielded a split-plot, repeated-measures design, allowing us to independently test for biotic versus abiotic (temperature and moisture) drivers of hysteretic behavior. The experiment lasted 7 months (4 months of establishment phase and 3 months of measurements).

### Continuous estimates of soil CO_2_ efflux

Building on the methods described by Barron-Gafford *et al*.^6^, we calculated *F*_*soil*_ in 30 minute increments using continuously operating solid-state CO_2_ sensors (GM222, Vaisala, Helsinki, Finland). Tang *et al*.^70^ provide a thorough description of the sensors operation. Briefly, each CO_2_ sensor is managed by a datalogger via a multiplexer. Holes on the bottom surface of the sensor allow CO_2_ to diffuse three-dimensionally through a membrane surrounding the probe. As described in detail by Pangle *et al*.^69^, we extracted discrete samples of the soil gas phase through gas-sampling tubes installed in the soil at three depths of 5, 20, and 50 cm. These tubes were constructed from 0.5-m length and 0.0064-m diameter microporous Teflon tubing with pore sizes ranging from 10 to 35 µm (Parker 1 103-0125031-NT-1000, Controlled Motion Solutions). That tubing was connected to non-porous tubing, sealed together with epoxy and heat shrink tubing. Gas-phase sampling was accomplished by using a flow-through loop linked in-line to a sealed CO_2_ probe housing (GMK220, Vaisala, Helsinki, Finland) with a GM222 probe inside. [CO_2_] at each depth was measured for two-minute-period every 20 minutes. The probe was flushed between each measurement. [CO_2_] readings were corrected for temperature and pressure using data collected by co-located sensors.

*F*_*soil*_ was calculated according to the “gradient method” using Fick’s first law of diffusion^27,30–33,49,52,70–72^, as modified by Sanchez-Cañete *et al*.^19^. In previous studies^6,39,52^, the daily degree of hysteresis was calculated as the difference between maximum and minimum *F*_*soil*_ for the daily median temperature. In our experiment, the presence of a near-constant soil temperature treatment makes the use of daily median temperatures less useful. We calculated instead a daily, microhabitat-specific amplitude of hysteresis as the difference between maximum and minimum *F*_*soil*_ for the entire day.

### Leaf-level measurements of photosynthetic activity

Rates of photosynthetic CO_2_ assimilation (*A*_*net*_) were measured on twelve *P. velutina* and twelve *B. curtipendula* individuals using a portable gas-exchange system (LI-6400; LI-COR, Lincoln, Nebraska, USA), which allows the user to create a stable microenvironment inside the cuvette that mimics ambient conditions outside. Following the procedures described by Barron-Gafford *et al*.^73^, *A*_*net*_ measurements were made continuously for a 24-hour period with the 12-hour day length. We used the LI-6400 red-blue light source (LI-6400-02b) to mimic the local levels of irradiance. Once sealed into the chamber, the leaf was acclimated to a CO_2_ setpoint of 400 ppm, the ambient air temperature, the ambient relative humidity, and a constant flow rate of 700 µmol s^−1^. Leaves placed into the cuvette were allowed to acclimate to current conditions and stabilize for a minimum of 30 minutes prior to the first gas exchange measurements. The portable photosynthesis system was then set on an auto-log procedure to match current temperature and relative humidity levels, acclimate the leaf, match the internal infrared gas analyzers, and log *A*_*net*_ upon reaching a steady value every 30 minutes. Within each species, all measures were conducted on intact leaves of similar size; we selected leaves of like age – the most recent, fully unfurled leaf. This procedure for measurements of rates of *A*_*net*_ was repeated across both temperature treatments and both wet and dry soil conditions for the three vegetated mesocosms to capture a spectrum of physiological activity, for a total of 72 individual diel measurements. Leaves were cut after each measurement to be scanned. Their area was determined using the Image J software (Schneider, Rasband & Eliceiri, 2012), allowing to calculate A_*net*_ per surface area.

### Statistical analysis

Data analyses were performed using the R statistical software (version 3.5.1; R Core Team, 2018). Mixed effect linear models were fitted to analyse the effects of the treatments on the hysteresis amplitude and *A*_*net*_ (nlme package; Pinheiro *et al*. 2015). The data fulfilled the heteroscedasticity and normality conditions necessary to fit linear models. The experimental cells were considered as random factors in both models. The models were simplified based on the Akaike Information Criterion. For the analysis of hysteresis amplitude, the soil temperature treatments (fluctuating vs. constant), the soil moisture treatments (wet vs. dry) and the vegetation type (mesquite, grass, mixture, or bare soil) were defined as fixed factors. The model fitted for the *A*_*net*_ analysis was similar, but instead of considering 4 vegetation types, we considered only mesquite and bunchgrass. In order to focus on the general effect of plant species, only monocultures were taken into account for *A*_*net*_. We used the *A*_*net*_ values from 11:30 to 19:30 to ensure we covered most of the daily patterns. To take into account that ecosystem functioning could change over time, the different ‘two week periods’ of measurements were included as fixed factors in models. Post-hoc pairwise comparisons were calculated from the models using the adjusted mean and Tukey-Kramer method (*lsmeans* package; Lenth 2018). The r.squaredGLMM function (MuMIn package; Barton 2018) was used to calculate marginal and conditional model R^2^ such as obtaining the part of variance explained by fixed factors and random effect, respectively (Nakagawa & Schielzeth, 2013).

To analyse how the hysteresis amplitude was affected by soil water content, *A*_*net*_ and *F*_*soil*_, person correlation analysis was used to calculate correlation coefficients. Because there is a single hysteresis amplitude value per day, we selected the value of volumetric water content, *A*_net_ and *F*_*soil*_ at mid-day (15:30) to test the correlation.

## Supplementary information

Supplementary information
