# Author Correction: Biotic soil-plant interaction processes explain most of hysteretic soil CO_2_ efflux response to temperature in cross-factorial mesocosm experiment

**DOI:** 10.1038/s41598-020-60084-5

**Published:** 2020-02-20

**Authors:** Yann Dusza, Enrique P. Sanchez-Cañete, Jean-François Le Galliard, Régis Ferrière, Simon Chollet, Florent Massol, Amandine Hansart, Sabrina Juarez, Katerina Dontsova, Joost Van Haren, Peter Troch, Mitchell A. Pavao-Zuckerman, Erik Hamerlynck, Greg A. Barron-Gafford

**Affiliations:** 1 Centre de recherche en écologie expérimentale et prédictive (CEREEP-Ecotron IleDeFrance), Département de biologie, Ecole normale supérieure, CNRS, PSL University, 77140 St-Pierre-les-Nemours, France; 2 Departamento de Física Aplicada, Universidad de Granada, 18071 Granada, Spain; 3 Sorbonne Université, CNRS, Institut d’Écologie et des Sciences de l’Environnement de Paris (iEES-Paris), Faculté des Sciences et Ingénierie, 75005 Paris, France; 4 Institut de Biologie de l’Ens (IBENS), Département de biologie, Ecole normale supérieure, CNRS, PSL University, 75005 Paris, France; 5 Department of Ecology and Evolutionary Biology, University of Arizona, Tucson, Arizona 85721 United States; 6 Biosphere 2, Office of Research, Development, & Innovation, University of Arizona, Tucson, Arizona 85721 United States; 7 Department of Hydrology & Atmospheric Sciences, University of Arizona, Tucson, Arizona 85721 United States; 8 Department of Environmental Science and Technology, University of Maryland, College Park, Maryland 20742 United States; 9 US Department of Agriculture-Agricultural Research Service, Eastern Oregon Agricultural Research Center, Burns, OR 97720 United States; 10 School of Geography & Development, University of Arizona, Tucson, Arizona 85721 United States

Correction to: *Scientific Reports* 10.1038/s41598-019-55390-6, published online 22 January 2020

The original version of this Article contained an error in the title of the paper, where the word “hysteretic” was incorrectly given as “hysteric”. This has now been corrected in the PDF and HTML versions of the Article and in the accompanying Supplementary Information file.

