# Supplementary material for: Biotic soil-plant interaction processes explain most of hysteric soil CO2 efflux response to temperature in cross-factorial mesocosm experiment
Source: Sci Rep. 2020 Jan 22;10:905. doi: 10.1038/s41598-019-55390-6 (PMC6976568; doi:10.1038/s41598-019-55390-6)

Supplementary Information for:

Biotic soil-plant interaction processes explain most of hysteric soil CO_2 efflux response to temperature in cross-factorial mesocosm experiment

Yann Dusza, Enrique P. Sanchez-Cañete, Jean-François Le Galliard, Régis Ferrière, Simon Chollet, Florent Massol, Amandine Hansart, Sabrina Juarez, Katerina Dontsova, Joost van Haren, Peter Troch, Mitchell A. Pavao-Zuckerman, Erik Hamerlynck, and Greg A. Barron-Gafford

Supplementary Method 1

General overview of the experimental set-up. Upper left, schematic representation of the four mesocosms with distinct plant-soil communities and photographs (small insets) of the soil mesocosms, plasma lighting system and soil CO2 measurement? Upper right, workflow for data acquisition including (1) a Pyrocontrol system to acquire data from gaz sensors (GM), soil WC (MAS) and temperature (PT100) as well as atmospheric pressure (PRESS) and (2) an acquisition system to combine raw data from sensors with data from light, watering and weather conditions into a DB server for subsequent analyses. Lower left, photograph of the 4 mesocosms at the early stages of plant growth during the establishment phase. Lower right, detailed photograph illustrating the set-up used to measured soil CO2 efflux. The homemade set-up includes 3 CO2 sensors, a manifold to connect with up to 12 gas loops in soils of 4 mesocosms at 3 different depths, a flushing system and power supplies.

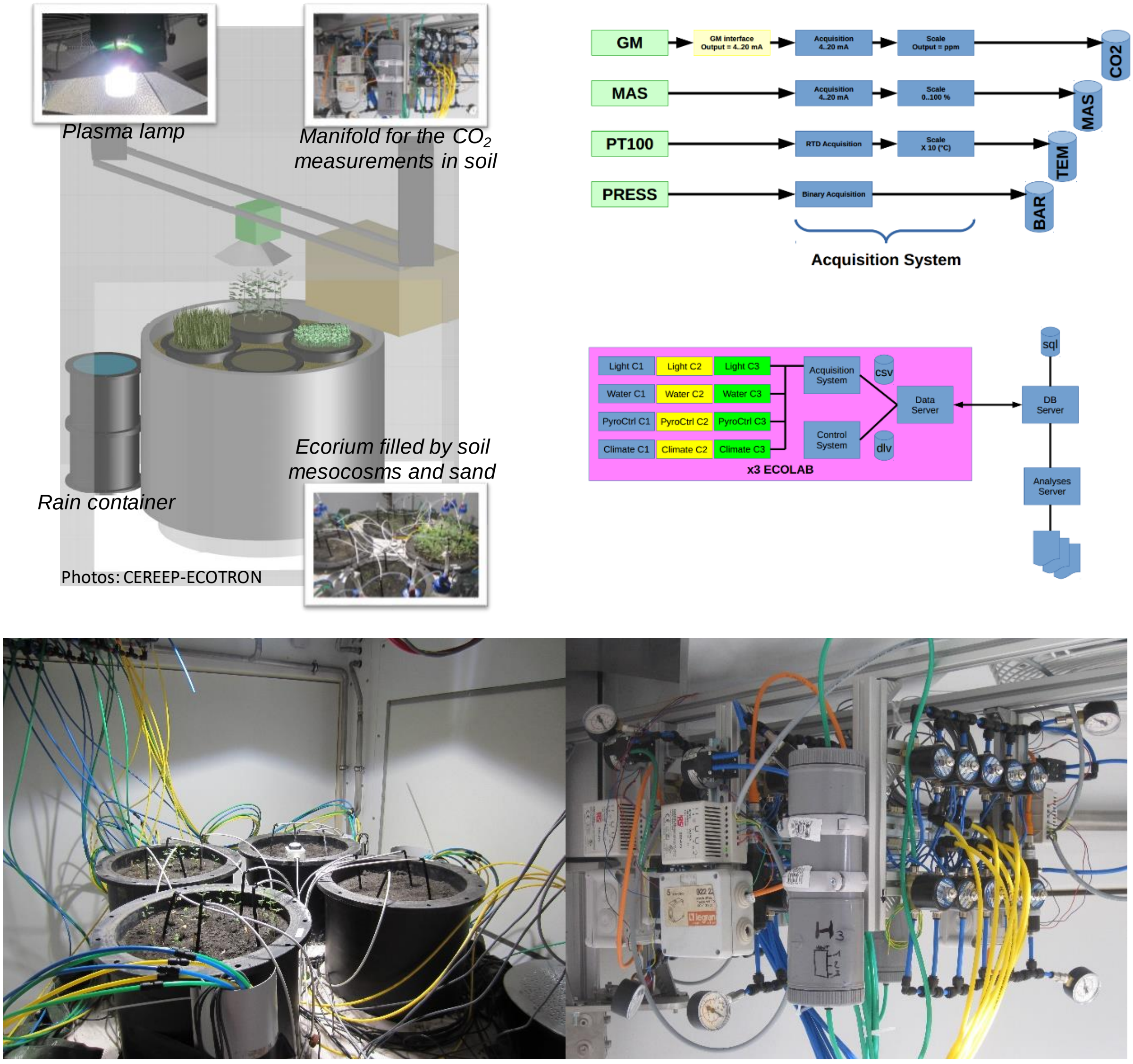

Supplementary Table 1

Aboveground and belowground mean biomass (± standard errors) for Grasses and Mesquites grown in monoculture. Mixtures are not represented due to the difficulty to separate plant roots.

	Aboveground (g)	Belowground (g)
Grass	26.39 ± 3.70	24.15 ± 6.49
Mesquite	4.10 ± 2.39	2.65 ± 1.30

Supplementary Figure 1

Average diel soil CO_2 efflux plotted against average soil temperature for all biotic treatments within soil temperature / irrigation treatments. Lights were turned on at 09:30 (represented by a triangle) and shut off at 21:30 (represented by a square). Arrows represent the clockwise hysteresis detected in all situations. F_{soil} was estimated from 5cm depth to the surface.

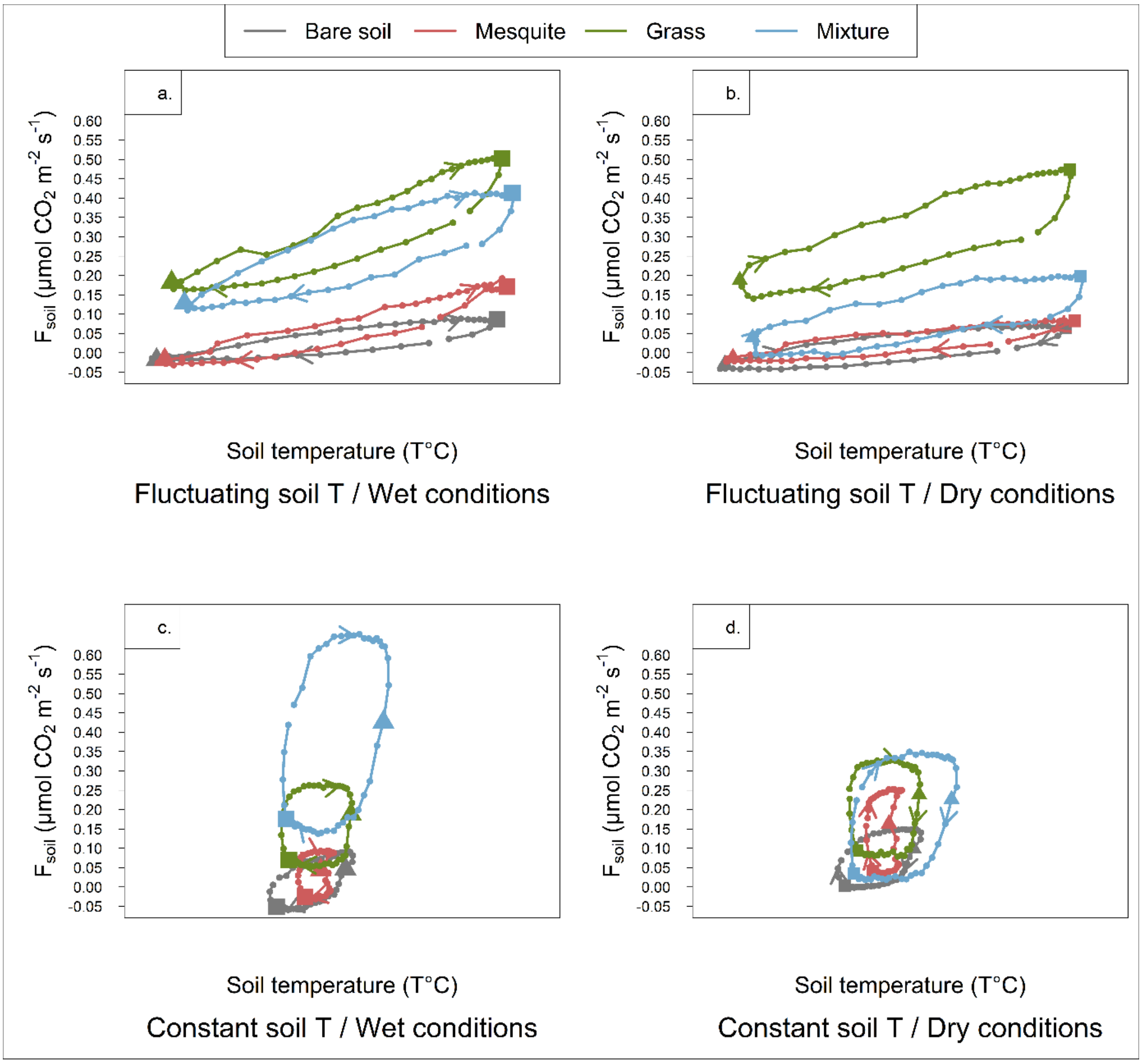

Supplementary Figure 2

Average diel soil CO_2 efflux (F_{soil}) plotted against soil temperature. For soil CO_2 efflux between 20 and 5 cm ($F_{soil\ 5\text{-}20}$), the 20 cm soil temperature was plotted. For $F_{soil\ 20\text{-}50}$, the 50 cm soil T was plotted. Scales are the same as for Figure 1.

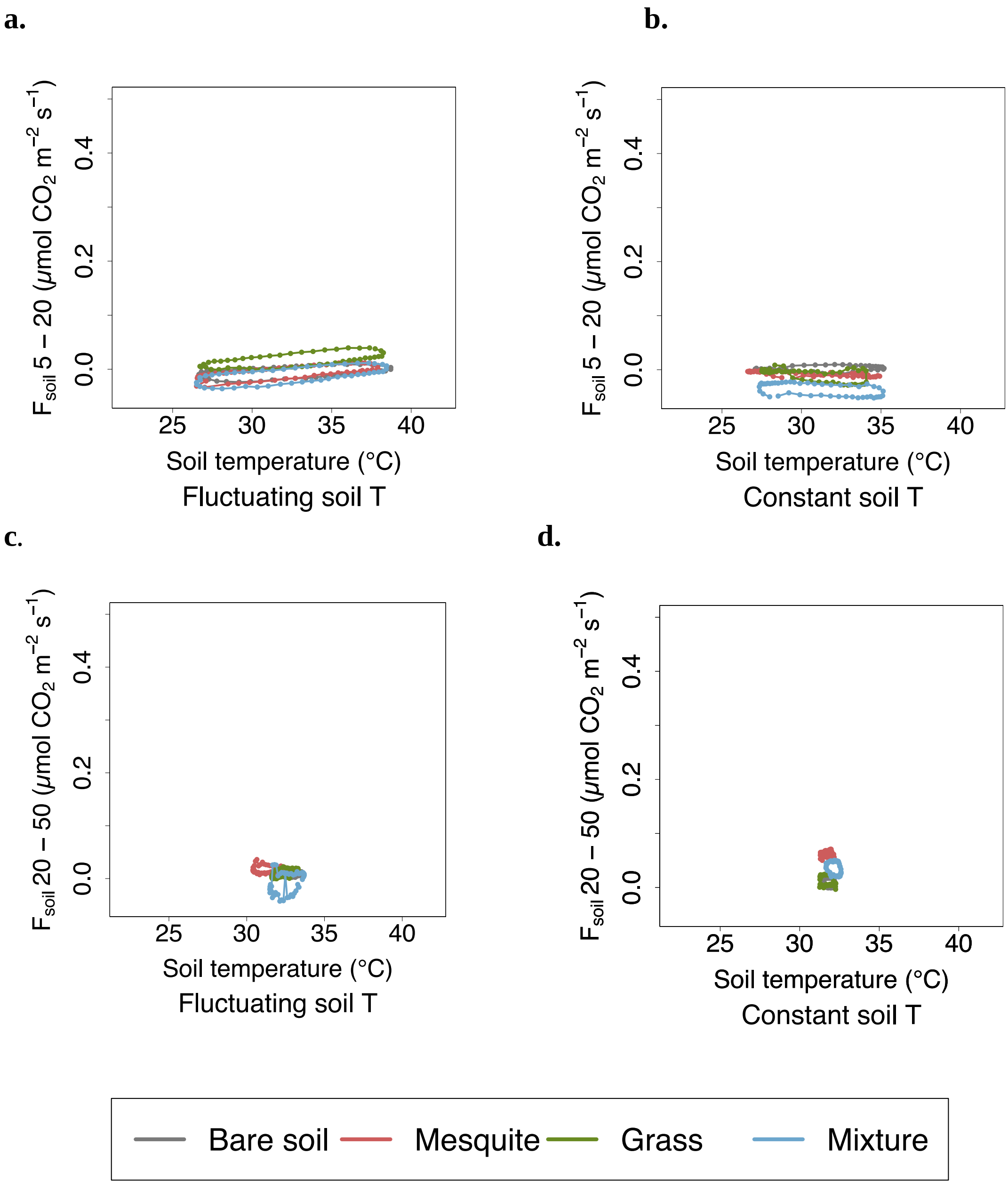

Supplement: Supplementary file 1 — Supplementary information [file 41598_2019_55390_MOESM1_ESM.pdf]
